# Associated factors for the utilization of institutional delivery services in Nepal: Findings from the Nepal Demographic Health Survey, 2022

**DOI:** 10.1371/journal.pone.0322309

**Published:** 2025-05-08

**Authors:** Om Chandra Thasineku, Sudesh Pandit, Devaraj Acharya, Yogendra Bahadur Gurung

**Affiliations:** 1 Research Centre for Educational Innovation and Development [CERID], Tribhuvan University [TU], Balkhu, Kathmandu, Nepal; 2 Prithvi Narayan Campus, Tribhuvan University, Pokhara, Nepal; 3 Central Department of Population Studies, Tribhuvan University [TU], Kirtipur, Kathmandu, Nepal; Patan Academy of Health Sciences, NEPAL

## Abstract

**Introduction:**

Institutional delivery provides skilled obstetric health care, postnatal care, and essential medical timely intervention to enhance the health of mothers and children. In Nepal, the proportion of institutional deliveries has increased from 8 percent in 1996–79 percent in 2022, although it is not satisfactory. This study investigates disparities in the utilization of institutional delivery service across associated factors related to residential factors, socio-economic factors, health service-related factors, and bio-demographic factors.

**Methods:**

We used secondary data from the Demographic and Health Survey (DHS) 2022 of Nepal. It involves a sample of 1977 eligible women aged 15–49 who had given birth within two-year preceding the survey. We considered institutional delivery as an outcome variable, while residential, socio-economic, bio-demographic, and health service-related factors as independent variables. Descriptive analysis and binary logistic regression analysis for crude and adjusted odds ratios (AOR) along with 95% confidence interval (CI) were utilized.

**Results:**

Of the total 1977 women, 1569 (79.4%) opted for institutional delivery. Women belonging to the Muslim ethnic group had lower odds (AOR:0.500, 95% CI: 0.259–0.966, p < 0.050) compared to their reference group. Similarly, the likelihood of opting for institutional delivery was significantly lower among women who required more than 30 minutes time to reach a health facility (AOR:0.626, 95% CI: 0.491–0.800, p < 0.001) and those having 6 + parity (AOR:0.080, 95% CI: 0.032–0.205, p < 0.001) compared to their reference group. In the contrary, women from Terai region (AOR:2.428, 95% CI: 1.194–4.937, p < 0.050), Bagmati Province (AOR:2.327, 95% CI: 1.179–4.593, p < 0.050), secondary and higher education level (AOR:3.161, 95% CI: 2.141–4.668, p < 0.001), richest wealth group (AOR:13.451, 95% CI: 5.231–34.589, p < 0.001), and antenatal care (ANC) visits 4 and more times (AOR:5.084, 95% CI: 2.7963–9.242, p < 0.001), were noticed more likely to choose for institutional delivery compared to their reference group, respectively.

**Conclusion:**

The result shows the ecological region, province, ethnic group, distance to reach health facility, parity, respondents’ education level, wealth index and ANC visits, and mother’s age in 5-year groups are the associated factors for the utilization of institutional delivery service in Nepal. It highlights the need for targeted interventions to enhance the utilization of institutional delivery services. Addressing socio-economic and geographical disparities, economic barriers, advancing education, promoting antenatal care visits, and ensuring nearer healthcare accessibility are crucial to achieving the equitable maternal and neonatal health care through institutional delivery in Nepal. It is concluded that more attention needs to be paid to areas where the severity persists by professionals and policymakers as well.

## Introduction

Globally, nearly 800 women died every day due to preventable causes related to pregnancy and childbirth in 2020 [1,2]. Shockingly, a maternal death occurred approximately every two minutes [2,3]. Of all maternal deaths, approximately 99% occurred in developing countries [4]. Nearly 95% of maternal deaths befallen low and lower-middle-income nations in 2020 [1]. Most maternal deaths can be prevented through prompt intervention by skilled healthcare professionals within a supportive environment [5] in institutional delivery. Prior childbirth experiences, health care provider’s behavior and perceived quality of care can increase the likelihood of choosing institutional place of delivery [6–9].

Institutional delivery service is an evidence-based intervention to decrease maternal deaths by providing safe births, minimizing actual and potential complications, and improving survival rates for both mothers and newborns [10,11]. However, in developing countries, most deliveries occur at home without the presence of skilled birth attendants [11]. Globally, the rate of institutional deliveries increased from an average of 51% in 2000 to over 76% in 2015 [12]. In Nepal, the percentage of institutional deliveries has increased over time, from 8% in 1996 to 64% in 2016 (lower than the global average) and 79% in 2022 [13]. While many countries have prioritized achieving 100% of deliveries in institutions as their primary strategy for minimizing maternal mortality [14].

Increasing the proportion of institutional delivery plays a vital role in lowering health risks for both mothers and babies. Appropriate medical care and hygienic conditions during delivery can lower the possibility of complications and infections, thereby reducing morbidity and mortality rates for both mothers and babies [15].Institutional delivery with skilled birth attendants is a central focus of initiatives aimed at lowering maternal and infant mortality rates [16]. Globally, from 2015 to 2021, approximately 84% of births were assisted by skilled health professionals, which includes medical doctors, nurses, and midwives [17]. The global aim is to achieve a 90% average coverage of births attended by skilled health personnel worldwide, while at the national level, the target is for 90% of countries to attain over 80% coverage [18].

The government of Nepal initiated demand side intervention in maternal health with the aim of maternity incentive Scheme in 2005 to enhance institutional delivery. This scheme aimed to share the cost of transportation to health facilities. In 2009, the Aama Programme was introduced, which not only provided transportation incentives but removed user fees for all delivery care services [19]. Financial incentives help to reduce barriers to institutional delivery, evidence suggests disparities persists, particularly in rural and poor population. Nonmedical factors like convenience and socio cultural norms also influence the health care facility utilization [20].

As per Sustainable Development Goal 3.1, the aim by 2030 is to decrease the global maternal mortality ratio to below 70 per 100,000 live births [2], while ensuring that no nation’s maternal mortality ratio exceeds double the global average [21]. Nepal has committed to decrease its maternal mortality ratio (MMR) from 281 per 100,000 live births in 2006–116 by 2022, 99 by 2025, and 70 by 2030 [21]. Nepal has achieved impressive advancements in lowering its maternal mortality rate (MMR), declining from 850 deaths per 100,000 live births in 1990–258 deaths per 100,000 live births in 2015 [22]. Furthermore, the MMR is estimated 151 per 100,000 live births in Nepal [21], and the study also revealed inequalities in MMR between provinces.

Nepal needs to speed up to accelerate advancements in the utilization of maternal health services along with institutional deliveries to meet SDG targets for reducing maternal mortality. Despite promising national averages, it is essential to recognize associated factors regarding the disparities in the utilization of institutional delivery service across different residential factors, socio-economic factors, health service-related factors, and bio-demographic factors.

## Methods

### Study design

This study deployed quantitative research design and utilized secondary data obtained from the Nepal Demographic Health Survey [NDHS] 2022. The NDHS is a nationally representative survey that provides periodically updates about demographic and health characteristics of the people of Nepal [23]. The survey was carried out by New ERA under the supervision of the Ministry of Health and Population (MoHP), was technically assisted by UCF International [13]. Details of the methodology can be obtained from the NDHS 2022 report [13].

### Study setting

The study setting of NDHS 2022 was carried out with the territory of Nepal. Since 2015, the administrative structure of Nepal has been organized into three levels of government: the federal government, seven provincial governments, and 753 local governments. There are 6 metropolitan cities, 11 sub-metropolitan cities, 276 urban municipalities, and 460 rural municipalities under local administration [24].

### Sample and sampling

The sampling frame for the 2022 NDHS is an updated version of the 2011 Nepal Population and Housing Census (NPHC) provided by the National Statistical Office [13]. The NDHS 2022 survey included samples from all seven provinces and three ecological regions of Nepal, representing national representativeness. Before sample selection, NDHS 2022 involved stratifying all seven provinces into urban and rural areas, resulting in 14 sampling strata from each province. The NDHS 2022 utilized two stages of a stratified sampling approach. Using the probability-proportional-to-size method, 476 primary sampling units (PSUs) were chosen in the first stage, with 248 from urban areas and 228 from rural areas. 30 households were chosen from each PSU in the second stage, resulting in a total sample size of 14,280 households, with 7,440 from urban areas and 6,840 from rural areas. Out of the surveyed households, 15,238 women aged 15–49 were eligible women for the NDHS survey. Of these 14,845 eligible women were successfully interviewed, yielding a response rate of 97.4 percent. In order to address the objectives of the study, we analyzed the 1977 eligible women aged 15–49 with a live birth in the two years preceding the survey.

### Data collection and processing

The data collection for the survey was conducted from January 5, 2022 to June 22, 2022. After the finalization of all questionnaires in English, they were translated in the language: Nepali, Maithili, and Bhojpuri. Four types of survey tools: the Household Questionnaire, the Woman’s Questionnaire, the Man’s Questionnaire, and the Biomarker Questionnaire were utilized in the NDHS 2022. To facilitate computer-assisted personal interviewing (CAPI), the system was programmed into tablet computers with the capability to choose from any of three languages. Tablet computers were used to gather data by the enumerators. Nepal Health Research Council (NHRC) and the ICF Institutional Review Board evaluated the survey protocol. Nineteen teams were employed for data collection. One supervisor, one male interviewer, one biomarker specialist, and three female interviewers were included in each team. After the completion of data collection in each cluster, the data files were registered and examined for incompleteness, inconsistencies, and outliers. Inconsistencies and errors were promptly reported to the field team for review in order to minimize issues moving forward.

### Outcome variable

The outcome variable of this study was the institutional delivery from the category of place of delivery. The variable “place of delivery” was recoded into a binary variable (yes/no) to represent institutional place of delivery for the calculation of binary logistic regression. Institutional delivery involved government hospital, PHC/primary hospitals, health post, basic health care center, urban health center, public sector, community health unit, private hospital, private clinic, other private and medical sector, FPAN, Merie Stopes, and NGO other, which were merged into a category labeled “yes” for institutional place of delivery. Respondent’s home, other home, and other were merged into “no” for institutional place of delivery.

### Independent variables

In this study, there were 14 independent variables which were categorized into 4 groups. The first group, residential factors, included place of residence, ecological zone, and province. The second group, socioeconomic factors, encompassed ethnic group, education level, education level of husband/partner, wealth index. The third group, comprised health service-related factors, included times to reach the nearest healthcare facility, visited health facility last 12 months, and frequency of ANC visits and lastly, bio-demographic factors included mother’s age in 5-year groups, duration of pregnancy, birth order, and pregnancy outcome. Among them, place of residence, ecological zone, province, sex of the household head, wealth index, visited health facility last 12 months, and pregnancy outcome independent variables were used as received, while the rest of independent variables were recoded into adjusted categories. These variables were adjusted to make the data more reader-friendly (Table 1).

**Table 1:** List of independent variables with adjusted category.

Variables	Category	Adjusted category
Ethnic group	Hill Brahmin and Hill Chhetri,	Brahmin/Chhetri
	Hill Dalit and Terai Dalit	Dalit
	Newar, Hill Janajati, Terai Janajati	Janajati
	Terai Brahmin/Chhetri, Other terai caste	Madhesi
	Muslim	Muslim
Education level of mother and Husband/partner	No education	No education
	Lover basic education (1–5) and upper basic education (6–8)	Basic
	Lower secondary (9–10), higher secondary (11–12) and more than secondary (13 and above)	Secondary and Higher
times to reach the nearest healthcare facility	0-29 minutes	Less than 30 minutes
	30-450 minutes	More than 30 minutes
Number of antenatal visits during pregnancy	No antenatal visits	None
	1-3	1-3
	4-20	4 and more
Mother’s age in 5-year groups	15-19	<20 years
	20-24, 25-29, 30-34	20-34
	35-39, 40-44, 45-49	35- 49
Duration of pregnancy in months	6,7,8	Less than 9 months
	9	9 months
	10	More than 9 months
Birth order number	1	1
	2,3	2-3
	4,5	4-5
	6,7,8,9	6 and more

### Statistical analysis

We utilized Stata 13.0 for data analysis [25]. Variables were presented in terms of frequency and percentage with respect to both total and institutional place of delivery separately. Complex sample analysis has been performed using Stata 13.0 with “svyset” and “svy” commands. This method accounts for the survey’s stratification, sampling weights, and clustering, ensuring accurate estimates and valid significance tests. Both univariate and multivariate binary logistic regression analyses were performed to find the relationship between institutional delivery and various factors (residential factors, socio-economic factors, health service-related factors, and bio-demographic factors). Descriptive analysis was performed to summarize the characteristics of the sample population. Before the binary logistic regression test, we assessed the variance inflation factors (VIF) for the multicollinearity test and found that there were no multicollinearity issues among the variables within each group of independent variables [26]. The results of binary logistic regression analysis were presented in terms of crude odds ratio (COR) and adjusted odds ratio (AOR) at a 95% of confidence interval (CI). The COR is calculated independently for each variable, assessing the individual association with the outcome variable. On the contrary, AOR is calculated by considering multiple variables simultaneously among the group of four factors mentioned factors separately, accounting for potential confounding effects.

### Ethical approval

The DHS Program provided dataset access for this study. The survey protocol was reviewed and approved by the institutional review board of ICF and Nepal Health Research Council [13].

## Results

Table 2 outlines the distribution of women aged 15–49 who had live births in institutional place of delivery within the two years preceding the survey. Out of 1,977 women surveyed, 1569 (79.4%) women opted for institutional place of delivery. Of these women, a larger proportion addressed from urban areas (1,046, representing 80.9%) compared to rural areas (522, accounting for 76.5%). Regarding ecological region, the hill region had the highest percentage of institutional deliveries (529, or 81.6%). When considering provinces, Bagmati Province showed the highest (264, or 88.3%), while the Madhesh Province displayed the lowest (343, or 66.8%).

**Table 2:** Characteristics of women aged 15-49 who had a live birth with in the two years preceding the survey (n = 1977).

Characteristics		Frequency	Percentage	
Institutional delivery	Yes	1569	79.4	
	No	408	20.6	
		Frequency	ID Frequency	ID Percentage
**Residential factors**				
Place of residence	Urban	1294	1046	80.9
	Rural	683	522	76.5
Ecological region	Mountain	132	100	75.3
	Hill	648	529	81.6
	Terai	1196	940	78.6
Province	Koshi	368	300	81.5
	Madhesh	514	343	66.8
	Bagmati	299	264	88.3
	Gandaki	117	102	87.7
	Lumbini	335	283	84.4
	Karnali	152	110	72.4
	Sudurpaschim	191	166	86.8
**Socio-economic factors**				
Ethnic group	Brahmin/Chhetri	502	436	86.9
	Dalit	373	262	70.1
	Janajati	605	505	83.3
	Madhesi	357	272	76.2
	Muslim	136	92	67.3
	Other	2	2	100.0
Education level	No education	366	218	59.5
	Basic	677	501	74.0
	Secondary and higher	933	850	91.1
Education level of husband/partner	No education	188	115	61.3
	Basic	777	587	75.6
	Secondary and higher	956	833	87.1
	Don’t know	40	21	53.1
Wealth index	Poorest	444	292	65.8
	Poorer	443	325	73.3
	Middle	388	309	79.6
	Richer	395	344	87.1
	Richest	306	299	97.6
**Health service-related factors**				
Times to reach to nearest healthcare facility	Less than 30 minutes	1441	1179	81.8
	More than 30 minutes	536	390	72.7
Visited health facility last 12 months	No	181	124	68.8
	Yes	1796	1445	80.4
Frequency of ANC Visits	None	52	25	48.2
	1-3	324	201	62.2
	4 and more	1554	1307	84.1
	Don’t know	2	2	100.0
**Bio-demographic factors**				
Mother’s age in 5-year groups	<20 years	221	177	80.0
	20-34 years	1649	1308	79.4
	35- 49 years	107	83	78.1
Duration of pregnancy	Less than 9 months	222	186	83.8
	9 months	1729	1364	78.9
	More than 9 months	25	19	76.1
Birth order (Parity)	1	814	734	90.1
	2-3	980	731	74.6
	4-5	159	90	56.7
	6 and more	24	14	58.6
Pregnancy outcome	Most recent live birth	1932	1535	79.4
	Prior live birth	45	34	76.3
Total		1977	1569	79.4

*Note: 45 respondents are missing for ANC visit in data file.*

*ID: Institutional Delivery*

*ID Percentage: Row percent on the basis of frequency*

While considering socio-economic factors, in terms of ethnic group, Brahmin/Chhetri hailed the highest institutional delivery (436, or 86.9%), whereas Muslim hailed lowest (92, or 67.3%). However, there was a notable increasing trend observed concerning the education levels of women and their husbands/partners. This trend indicated an increasing tendency to opt for institutional delivery in ascending order, from those with no education to those with secondary and higher education levels. A similar increasing trend was also found in the wealth index, with a progression from the poorest category (292, or 65.8%) to the richest category (299, or 97.6%), indicating a higher likelihood of being selected for institutional delivery as wealth increases.

During the analysis of health service-related factors, there was a notable decreasing trend observed concerning the time to reach the nearest healthcare facility for opting for institutional delivery. This trend ranged from less than half an hour (1179, or 81.8%) to more than 30 minutes (390, or 72.7%). In contrast, there is an increasing trend observed in the frequency of antenatal visits, particularly among those who attended four or more visits (1307, accounting for 84.1%), compared to those who had no antenatal visit (25, representing 48.2%).

While analyzing, bio-demographic factors, a clear decreasing trend was observed in the likelihood of opting for institutional delivery. This trend was noticeable across mother’s age in 5-years groups, duration of pregnancy, birth order number, and pregnancy outcome. For the mother’s age in 5-year groups, institutional delivery rate was higher among younger age group mothers (<20 years: 177, or 80.0%) than older age group mothers (35–39: 83: 78.1%). Similarly, for duration of pregnancy, institutional delivery was higher in less than 9 months (186, or 83.8%) compared to more than 9 months (19, or 76.1%). Regarding the birth order, the first child had the highest institutional delivery rate (734, or 90.2%) than sixth and more (14, or 58.3%). Furthermore, pregnancy outcome was more recent live birth (1535, or 79.4%) than prior live birth (34, or 76.3%).

Table 3 presents the results of statistical tests for examining the associated factors for the utilization of institutional delivery services in Nepal. These results are based on data from nationally representative data, NDHS conducted in 2022. The analysis in this study employs binary logistic regression to identify the associated factors linked to the likelihood of having an institutional delivery. It provides both crude odds ratios (COR) and adjusted odds ratios (AOR), along with 95 percent confidence intervals (CI) and p values for selected independent variables.

**Table 3:** Associated factors for the utilization of institutional delivery.

Variables	Crude Odds Ratio (95% CI)	Sig.	Adjusted Odds Ratio (95% CI)	Sig.
**Residential factors**				
**Place of residence**				
Urban	Ref.		Ref.	
Rural	0.770(0.570-1.041)		0.790(0.581 to 1.074)	
**Ecological region**				
Mountain	Ref.			
Hill	1.454(0.754-2.803)		1.281(0.685 to 2.399)	
Terai	1.208(0.631-2.312)		2.428(1.194 to 4.937) *	<0.050
**Province**				
Koshi	Ref.		Ref.	
Madhesh	0.456(0.289-0.719) **	<0.010	0.337(0.206-0.552) ***	<0.001
Bagmati	1.719(0.887-3.332)		2.327(1.179-4.593) *	<0.050
Gandaki	1.614(0.827-3.153)		2.064(0.966-4.409)	
Lumbini	1.230(0.671-2.253)		1.111(0.606-2.035)	
Karnali	0.595(0.344-1.030)		0.947(0.527-1.704)	
Sudur Paschim	1.487(0.849-2.604)		1.682(0.955-2.963)	
**Socio-economic factors**				
**Ethnic group #**				
Brahmin/Chhetri	Ref.		Ref.	
Dalit	0.353(0.233-0.536) ***	<0.001	0.720(0.469-1.104)	
Janajati	0.754(0.507-1.121)		0.963(0.647-1.434)	
Madhesi	0.483(0.309-0.754) **	<0.010	0.598(0.361-0.989) *	<0.050
Muslim	0.310(0.161-0.594) ***	<0.001	0.500(0.259-0.966) *	<0.050
**Education level**				
No education	Ref.		Ref.	
Basic	1.939(1.447-2.598) ***	<0.001	1.406(1.034-1.913) *	<0.050
Secondary and higher	6.931(4.867-9.869) ***	<0.001	3.161(2.141-4.668) ***	<0.001
**Education level of husband/partner**				
No education	Ref.		Ref.	
Basic	1.961(1.330-2.893) **	<0.010	1.120(0.718-1.748)	
Secondary and higher	4.247(2.742-6.579) ***	<0.001	1.140(0.675-1.926)	
Don’t know	0.714(0.310-1.648)		0.565(0.241-1.321)	
**Wealth index**				
Poorest	Ref.		Ref.	
Poorer	1.422(1.029-1.966) *	<0.050	1.634(1.149-2.323) **	<0.010
Middle	2.031(1.442-2.860) ***	<0.001	2.061(1.427-2.977) ***	<0.001
Richer	3.493(2.298-5.310) ***	<0.001	3.267(2.028-5.264) ***	<0.001
Richest	21.452(8.920-51.591) ***	<0.001	13.451(5.231-34.589) ***	<0.001
**Health service-related factors**				
**Times to reach to nearest healthcare facility**				
Less than 30 minutes	Ref.		Ref.	
More than 30 minutes	0.592(0.441-0.796) **	<0.010	0.626(0.464-0.845) **	<0.010
**Visited health facility last 12 months**				
No	Ref.		Ref.	
Yes	1.868(1.342-2.601) ***	<0.001	1.525(1.099-2.117) *	<0.050
**Frequency of ANC visits #**				
None	Ref.		Ref.	
1-3	1.766(0.945-3.298)		1.617(0.892-2.934)	
4 and more	5.672(3.059-10.519) ***	<0.001	5.084(2.796-9.242) ***	<0.001
**Bio-demographic factors**				
**Mother’s age in 5-year groups**				
<20 years	Ref.		Ref.	
20-34 years	0.961(0.699-1.380)		2.287(1.505-3.475) ***	<0.001
35- 49 years	0.888(0.484-1.629)		4.496(2.329-8.681) ***	<0.001
**Duration of pregnancy**				
Less than 9 months	Ref.		Ref.	
9 months	0.722(0.475-1.096)		0.844(0.555-1.283)	
More than 9 months	0.617(0.223-1.709)		0.644(0.221-1.871)	
**Birth order (Parity)**				
1	Ref.		Ref.	
2-3	0.322(0.238-0.436) ***	<0.001	0.246(0.176-0.344) ***	<0.001
4-5	0.143(0.092-0.222) ***	<0.001	0.096(0.060-0.154) ***	<0.001
6+	0.155(0.064-0.378) ***	<0.001	0.080(0.032-0.205) ***	<0.001
**Pregnancy outcome**				
Most recent live birth	Ref.		Ref.	
Prior live birth	0.831(0.358-1.927)		0.516(0.227-1.176)	

* p < 0.050

** p < 0.010

*** p < 0.001

Ref.: Reference category

**#** Other from ethnic group and don’t know from frequency of ANC visits are not shown due to the nominal number of responses and cause inconsistent results, but they were included in the calculations.

Based on the result obtained from multiple variable analysis on the basis of different factors, women residing in rural areas exhibited a lower AOR of 0.790 (0.581 to 1.074 at 95% CI) for institutional deliveries compared to those in urban areas, indicating a trend towards lower odds of institutional deliveries in rural areas, although it was not significant. On the contrary, women who reside in the terai region area showed a higher AOR of 2.428 (1.194 to 4.937 at 95% CI, p < 0.050) for institutional deliveries compared to those in the mountain region, which was statistically significant. Similarly, women in Bagmati Province had a significantly higher AOR of 2.327 (1.179 to 4.593 at 95% CI, p < 0.050). Conversely, women living in Madhesh Province had a significantly lower AOR of 0.337 (0.206 to 0.552 at 95% CI, p < 0.001) for institutional deliveries compared to those in Koshi Province, which was statistically significant. These results exhibited significant disparities across the provinces in institutional delivery.

On the basis of output regarding ethnic group in socio-economic factors, Muslim ethnic group demonstrated half likelihood of AOR of 0.500 (0.259 to 0.966 at 95% CI, p < 0.050) in institutional deliveries compared to the Brahmin/Chhetri, which was statistically significant. Moreover, education level of women in secondary and higher showed the more than three-fold AOR of 3.161 (2.141 to 4.668 at 95% CI, p < 0.001) for institutional deliveries rather than that of women with no education, which was statistically significant. On the contrary, education level of husband/partner showed no significant difference with institutional delivery. Although it is also observed increased AOR in education level of husband/partner, but it was not statistically significant. A clear trend observed in wealth index, women from richest wealth group revealed more than thirteen-times higher AOR of 13.451 (5.231 to 34.589 at 95% CI, p < 0.001) for institutional deliveries compared to poorest wealth group, which was statistically significant.

While considering factors related to the health service-related factors, duration of more than 30 minutes time to reach the nearest healthcare facilities showed a reduced likelihood, with AOR of 0.626 (0.464 to 0.845 at 95% CI, p < 0.010) for institutional deliveries in reference to less than 30 minutes time to reach the nearest health facilities, which was statistically significant. Furthermore, an analysis of women who visited health facilities last 12 months showed a higher likelihood of AOR 1.525 (1.099 to 2.117 at 95% CI, p < 0.050) opting for institutional delivery rather than who didn’t visit health facilities last 12 months, which was statistically significant. In the same line, while analyzing women who attended antenatal care (ANC) visits four or more times, they demonstrated more than five-fold higher AOR of 5.084 (2.796 to 9.242 at 95% CI, p < 0.001) as compared to those who never attended ANC visits, which was also statistically significant.

While considering bio-demographic factors, age group 35–49 years of the mother’s age in 5-years groups exhibited more than four-fold heightened likelihood for institutional deliveries, with AOR of 4.496 (2.329 to 8.681 at 95% CI, p < 0.001) compared to mothers aged less than 20 years, and the difference was statistically significant. Moreover, according to birth order, women who had six and more birth order revealed the decreased likelihood with AOR of 0.080 (0.032 to 0.205 at 95% CI, p < 0.001) for institutional deliveries compared to first birth order, which was statistically significant.

## Discussion

We found that, despite the Aama programme offering free delivery service with transportation incentives, there remained disparity in the utilization of maternal healthcare services in institutional delivery services. The results emphasize the associated factors of residential, socio-economic, bio-demographic, and health service-related factors on the prevalence of institutional delivery in Nepal. The findings of the study illuminate the relationship among various factors associated with the decision to opt for institutional delivery in Nepal.

### Residential factors

Women living in rural areas revealed less tendency towards institutional delivery compared to those residing in urban areas aligned with the study [10,27,28]. Women in rural areas of Nepal are less likely to opt for institutional delivery due to lack of adequate health facilities, lack of easy access of transportation, lack of educational awareness, perception of poor quality of care, inadequate infrastructure in health facilities, and lack of skilled health personnel [22,29,30]. The odds of likelihood of institutional delivery were found to be higher in the hill and terai region when compared to the mountain region. The likelihood of institutional delivery was found to be lower in Madhesh Province [10] than in Koshi Province and twice as high in Gandaki and Bagmati Provinces.

The uneven utilization of institutional delivery services across provinces may be associated with disparities in both the availability and usage of maternal health services within those provinces [10,31]. Women in Madhesh Province are less likely to choose institutional delivery, even though there is largely plain and flat area, providing physical access to health services. Despite this, women in Madhesh Province are attributed by poor socio-economic conditions and socio-cultural beliefs, considering institutional delivery unnecessary unless complications occur [22]. Women from areas with less developed areas are less likely to utilize institutional delivery services compared to those from relatively more developed areas [29,32]. Addressing the issues, implementation of targeted interventions is essential for institutional delivery services among women residing in mountain regions, with a specific focus on Madhesh Province. Investing in infrastructure reduces travel time to health facilities, making it easier for women to access delivery services, and can encourage institutional delivery among women residing in regions with less developed area compared to those in more developed areas [29,32].

### Socio-economic factors

The findings of the study shed light on the involved interplay of socio-economic factors in influencing the choice of institutional delivery in Nepal. Firstly, analysis revealed notable disparities across ethnic groups. Muslim ethnic group exhibited the lowest prevalence of institutional delivery among other ethnic groups, probable causes such as lower levels of education and religious and cultural beliefs. While Brahmin/Chhetri ethnic groups demonstrated the highest rates compared to Janajati, Dalit and Madhesi ethnic groups. A similar study also reveals women from Brahmin/Chhetri ethnic group were observed to have a greater tendency to deliver their babies in health facilities [33,34]. Another study also yielded a similar result, indicating that women from less advantaged castes were less likely to deliver their babies in healthcare facilities [22]. Women from less advantaged ethnic groups are less tendency to utilize institutional delivery services compared to the relatively more advantaged ethnic groups. Ethnic differences were evident, with less likely among Muslim ethnic groups for institutional delivery, indicating the need for targeted interventions for Muslim ethnic groups.

Higher levels of education of women and her husband/partner also displayed a greater tendency towards institutional delivery compared to lowers level of education and people with no education. Some of studies also observed that there is a reduced likelihood of institutional delivery practice with a lower level of education among women [10,28,35] and husbands [33]. Similarly, educated women are more likely to deliver their babies in health facility compared to uneducated women [22,36]. So, education plays a crucial role in encouraging institutional delivery practices in Nepal. Educated women are more likely to possess the awareness, knowledge, and decision-making power necessary to prioritize institutional delivery. They understand the advantages of institutional delivery and are better equipped to navigate the healthcare system to access maternal healthcare services and contribute to reducing maternal and neonatal mortality rates in the country.

Women from the poorest wealth category revealed less likely to have institutional deliveries compared to the richest wealth category. A similar situation was also observed in the study, women from the poor wealth category were less likely to select institutional delivery in comparison to women from the richest wealth category [10,22,28], primarily due to the hidden costs associated with such deliveries (e.g., medicines, transportation) [37]. As a result, women from poorer households may notice institutional delivery as economically burdensome, leading them to choose alternative options such as home deliveries assisted by traditional birth attendants. This highlights the need to address financial obstacles and provide comprehensive support to ensure equitable access to institutional delivery services for all women, regardless of their socioeconomic status.

### Health service-related factors

Women are more likely to have institutional delivery if it takes less time to reach the nearest health care facility compared to those women who require more time to reach health facilities. Similarly, women who visited health facilities in the last 12 months were more likely to opt for institutional delivery than those who did not visit health facilities in the last 12 months. The distance between the residence and the institutional delivery facility has been identified as a major hindrance for women [30] in accessing and utilizing maternal health services, impacting both antenatal care (ANC) visits and institutional delivery [38]. This barrier affects both antenatal care visits and the choice of institutional delivery. So, initiatives aimed at reducing transportation barriers, improving infrastructure, and expanding healthcare facilities closer to communities can be supported to mitigate this challenge and ensure that all women have equitable access to essential maternal healthcare services.

Antenatal care is recognized as a pathway to institutional delivery [39]. A higher number of ANC visits demonstrated a higher tendency towards institutional delivery compared to those who never attended ANC visits. This result is in line with a study conducted in Ethiopia, a higher number of ANC visits tended to increase the utilization of institutional delivery services [29]. Same finding was also found in another study in Ethiopia, the finding that ANC attendance is associated with institution delivery [40]. Moreover, women who attended ANC for four or more times were observed to have greater tendency to give birth in an institutional delivery compared to those who attended less than four times [41,42]. The findings consistently indicate that a higher number of ANC visits are associated with an increased likelihood of opting for institutional delivery services. ANC as an entry point to maternal healthcare, serving not only to monitor pregnancy but also to educate and counsel women about the benefits of institutional delivery. Regular attendance to antenatal care (ANC), especially aiming for four or more visits, plays a vital role in promoting institutional delivery and enhancing maternal and child health outcomes.

### Bio-demographic factors

While examining bio-demographic factors, this study and similar research exhibited that mothers in higher age group were more likely to opt for institutional deliveries compared to mothers of lower age groups category [43,44]. Higher age category of mothers being more inclined towards institutional deliveries [10,28] due to awareness and concern about potential complications associated risks with advanced maternal age, prioritizing the safety and support provided by healthcare facilities during childbirth [45]. This study also assumes the same probable causes regarding the high age category of mothers.

The prevalence of institutional delivery among primiparous was notably higher [46], aligning with the findings of this study. Women with more parity were less likely to choose institutional delivery compared to those with fewer parity, despite the fact that risk for birth complications and maternal mortality is also high for women in higher parities [47]. It is also noted that the parity and institutional delivery are inversely correlated, which is consistent with present finding, i.e., women in first parity were more likely to deliver in a health facility compared to those in second or higher parity [48–50]. The inverse correlation between parity and institutional place of delivery is significantly influenced by previous childbirth experiences, perceptions of quality of care [6,8,9], and behavior of health care providers [7]. The reduced utilization of institutional delivery may be attributed to time and resource constraints faced by those managing larger families [39]. The study highlights a trend where primiparous women exhibit more likely to have institutional delivery, while higher-parity women show lower utilization, probably due to resource constraints.

## Strengths and limitations

This study employed data from a nationally representative survey that utilized standardized questionnaire and applied a multilevel modelling approach, providing reliable insight into key determinants and contributing significantly to the existing literature in Nepal. The data used in the study has many strengths in terms of sample size, sampling procedure, and data collection procedure. A cross-sectional study with a large sample size ensures the reliability of the statistical test for identifying the factors associated with institutional delivery in Nepal. This study addresses maternal health and institutional delivery services that can inform and aware policies to achieve SDG 3 (good health and well-being).

Self-reported information was collected retrospectively, which may raise the possibility of recall bias. We utilized secondary data, so some of the variables that might be associated with institutional delivery were missed in the study. Relying on secondary data limits the study’s ability to explore maternal perceptions and consent-based preference for home place of delivery. This study only highlights the association between variables but cannot explain causality.

## Conclusion

In conclusion, despite the provision of Aama Programme in selected hospitals of free delivery service and transportation incentives, disparities in the utilization of institutional delivery service exist in Nepal. Institutional delivery plays a crucial role in enhancing maternal and neonatal health outcomes to reduce maternal mortality and neonatal mortality, respectively. For the utilization of institutional delivery services in Nepal, the choice of delivery is influenced by various factors such as geographical region, ethnic group, education level, wealth index, time to reach nearest health facility, frequency of ANC visits, mother’s age in 5-year groups, and birth order (parity). It is crucial to address geographical and developmental imbalance, social disparities, economic constraints, promoting educational attainment, and enhancing healthcare accessibility with the required number of ANC visits for the utilization of institutional delivery services in Nepal. These are essential steps towards ensuring equitable access to maternal healthcare services and improving maternal and neonatal health outcomes through institutional delivery in Nepal. Policymakers should consider the findings while formulating strategies to overcome these challenges and enhance the utilization of institutional delivery services in Nepal.
